# Effect of Residual Insulin Secretion on Glycaemic Control Among Young Cameroonian Individuals With Type 1 Diabetes

**DOI:** 10.7759/cureus.104502

**Published:** 2026-03-01

**Authors:** Mesmin Dehayem Yefou, Marcelle Nourya Meli Ymelong, Martine Claude Etoa Etoga, Agoons Batakeh, Jean-Claude Katte, Charly Feutseu, Anne Boli, Eugene Sobngwi

**Affiliations:** 1 Department of Internal Medicine and Specialities, Faculty of Medicine and Biomedical Sciences, University of Yaoundé I, Yaoundé, CMR; 2 Endocrinology and Diabetes, Yaoundé Central Hospital, Yaoundé, CMR; 3 Internal Medicine, Woodhull Medical Center, NewYork, USA; 4 Department of Clinical and Biomedical Sciences, University of Exeter Medical School, Exeter, GBR; 5 Laboratory for Molecular Medicine and Metabolism, Biotechnology Center, University of Yaoundé I, Yaoundé, CMR; 6 Department of Internal Medicine, Faculty of Health Sciences, University of Bamenda, Bamenda, CMR

**Keywords:** glycaemic control, residual insulin secretion, stimulated c-peptide, type 1 diabetes, young cameroonian individuals

## Abstract

Objective

The objective of the article is to determine the effect of residual insulin secretion on glycaemic control among young Cameroonian individuals diagnosed with type 1 diabetes (T1D).

Methods

We conducted a hospital-based cross-sectional study from April to August 2022. Forty-two children and adolescents diagnosed with T1D attending the paediatric diabetes clinic of the Yaoundé Central Hospital in Cameroon were enrolled in the study. Residual insulin secretion was assessed by measuring plasma C-peptide concentrations before and after a mixed meal tolerance test (MMTT) using an enzyme-linked immunosorbent assay (ELISA) method. Glycosylated haemoglobin (HbA1c) was measured by boronate affinity chromatography with the HemoCue® HbA1c 501 analyser. Residual insulin secretion was classified as absent (<0.033 nmol/L), low (0.033-0.2 nmol/L), or high (≥0.2 nmol/L) based on stimulated C-peptide levels. Clinical characteristics and HbA1c values were compared between participants with high residual insulin secretion and the rest of the cohort.

Results

The median age of participants was 18.5 (16-24) years, with a median diabetes duration of three (2-10) years and a median HbA1c of 8.4% (6.9-11.3). Stimulated C-peptide was detectable (≥0.033 nmol/L) in 25 (60%) participants and above 0.2 nmol/L in 12 (29%). Compared with those without or with low residual insulin secretion, participants with high secretion had a shorter median duration of diabetes (two (IQR: 1-4) vs six (IQR: 2-11) years; p=0.009), lower median daily insulin doses (0.7 (IQR: 0.4-0.8) vs 0.9 (IQR: 0.7-1.3) IU/kg; p=0.008), fewer median episodes of severe hypoglycaemia (zero (IQR: 0-2) vs four (IQR: 1-6); p=0.006), higher median BMI (26.1 (IQR: 25-29.3) vs 22.9 (IQR: 19.4-25.6) kg/m²; p=0.02), and lower median HbA1c levels (7.1 (IQR: 6.1-8.3) vs 9.1 (IQR: 8.1-12.2)%; p=0.01).

Conclusion

High residual insulin secretion was observed in nearly one-third of young Cameroonians with T1D and was strongly associated with better glycaemic control.

## Introduction

Type 1 diabetes (T1D) is the most common form of diabetes in children and adolescents and results from autoimmune β-cell destruction, usually leading to absolute insulin deficiency [1]. Individuals with T1D require lifelong insulin therapy to prevent acute and chronic complications, as well as premature death [2]. Traditionally, it was believed that β-cell function was severely impaired at diagnosis, with complete loss of function shortly thereafter [3,4]. However, research has shown that many individuals with T1D retain varying levels of residual insulin secretion for years after diagnosis [4,5]. This persistence has been linked to better glycaemic control, fewer hypoglycaemic episodes, and a lower risk of microvascular complications [6,7]. Understanding these protective effects is important in every setting but becomes especially relevant in regions where access to insulin and structured diabetes care can be more challenging.

In Cameroon, as in many other sub-Saharan African countries, access to insulin for optimal T1D has been historically challenging. This recognition led to the pioneering of the Changing Diabetes in Children (CDiC) project, which has provided free diabetes care to young people living with T1D in Cameroon since 2010 [8], thereby establishing a large national cohort. Despite this support, many still struggle to achieve adequate glycaemic control. A study conducted early in the project found that those with better glycaemic control typically had shorter disease duration, fewer hypoglycaemic episodes, and lower insulin requirements, features suggestive of preserved residual endogenous insulin secretion [9]. Confirmation of such a hypothesis would provide arguments in favour of a systematic measurement of residual insulin secretion at the diagnosis of T1D in young subjects in Cameroon by measuring C-peptide, thus making it possible to identify subjects without or with low residual insulin secretion in whom support measures should be intensified to obtain the best glycaemic control and avoid acute and chronic complications. This study aimed to robustly assess endogenous insulin secretion in young Cameroonians living with T1D, and its impact on glycaemic control. 

## Materials and methods

Study design and participants

This was a hospital-based, cross-sectional study conducted between April and August 2022 at the Endocrinology and Metabolic Diseases Unit of the Yaoundé Central Hospital, Cameroon. Forty-two children and adolescents diagnosed with T1D, enrolled in the CDiC project, were recruited. Exclusion criteria included estimated glomerular filtration rate (eGFR) <60 mL/min/1.73 m², pregnancy, or breastfeeding. 

Study procedure

After obtaining informed consent and assent, participants completed a structured questionnaire on sociodemographic, diabetes history, and treatment. Weight, height, and blood pressure were measured using standardised procedures. Body mass index (BMI) was calculated and classified according to WHO criteria, including age-specific cutoffs for 5-19 years [10]. Body composition was assessed with a TANITA® SC-240MA analyser (Tanita Corporation, Tokyo, Japan). Fasting blood samples were collected for glycosylated haemoglobin (HbA1c), creatinine, and C-peptide. Participants then underwent a standardised 2-hour mixed meal tolerance test (MMTT).

The MMTT was performed in the morning between 8 and 9 AM, after an overnight fast, with no food after 10 PM the preceding day, and no rapid insulin injection for at least six hours. The meal test consisted of preparation containing 82.5 g of carbohydrates, 37.5 g of proteins, and 12 g of fats (i.e., 55% carbohydrate, 24% protein, and 21% fat) according to the composition of the test meal used in the Diabetes Control and Complications Trial (DCCT) study [11]. The ingredients used for the meal were ground flavoured rice, olive oil, and skimmed milk. The quantities were measured using a food scale, and warm water was added to the mixture up to a maximum of 360 ml. This mixture was ingested by the participant within five minutes. Venous blood samples were collected 90 minutes after the ingestion of the meal to measure post-prandial (stimulated) C-peptide concentrations.

Laboratory assessment of glycaemic control and endogenous insulin secretion

HbA1c was measured by boronate affinity chromatography using HemoCue® HbA1c 501 analyser (HemoCue, Angelhom, Sweden), with a limit of detection ranging from 4 to 14% and a coefficient of variation of 2.24% (low level) and 2.09% (high level). The analyser was factory calibrated and automatically self-adjusted during each power-up and during each assay; daily and monthly cartridge checks were run regularly for quality control. Serum creatinine was measured by the Jaffe kinetic method using reagents manufactured by Biolabo (Maizy, France), with a limit of measurement ranging from 4 to 150 mg/l, an intra-assay coefficient of variation of 1.8%, and an inter-assay coefficient of variation of 4.0%. Quality control was performed regularly for assay validation using normal and pathological sera control. Plasma C-peptide was determined by sandwich enzyme-linked immunosorbent assay (ELISA), using Sinothinker SK202 ELISA reader (Sinothinker, Schenzen, China) and Calbiotech ELISA reagents (Calbiotech, California, USA), with detection between 0.033-6.6 nmol/L. Residual endogenous insulin secretion was categorised into three groups based on plasma C-peptide: absent <0.033 nmol/L; low 0.033-0.2 nmol/L; high ≥0.2 nmol/L. The threshold for high C-peptide ≥0.2 nmol/L was chosen in reference to that used in the DCCT study [11].

Statistical analysis

The data collected from the questionnaire were entered into a pre-conceived Microsoft Excel sheet (Microsoft Corp., Redmond, USA) and doubly checked by two independent data assessors for consistency and accuracy. The data was then exported to and analysed using the Statistical Package for the Social Sciences (SPSS) Version 26 (IBM SPSS Statistics for Windows, Armonk, USA). Quantitative variables were expressed as medians (IQR) and qualitative variables as counts/percentages. Chi-square tested categorical variables; Mann-Whitney tested continuous variables. Spearman's correlation assessed associations. P-value <0.05 was considered statistically significant.

Ethical considerations

The study was conducted in accordance with the Declaration of Helsinki and was approved by the Ethical Review Board of the Faculty of Medicine and Biomedical Sciences, University of Yaoundé I (N°414/UY1/FMSB/VDRC/DAASR/). Written informed consent and assent were obtained from all the study participants. 

## Results

Characteristics of study participants

Of the 42 participants, 28 (67%) were female, and 24 (57%) were children/adolescents (age ≤19 years). Median age was 18 (IQR: 16-24) years, median BMI was 23.5 (IQR: 20.7-27.7) kg/m², and median diabetes duration was 3 (IQR: 2-10) years. Most had normal BMI (54%), while 5% were underweight. The median daily insulin dose was 0.8 (IQR: 0.7-1.3) IU/kg/day, and 36 (86%) participants were on ≥3 injections/day. The median HbA1c was 8.4% (IQR: 6.9-11.3), and 28 (67%) participants had poor glycaemic control (HbA1c>7%). The median number of self-reported hypoglycaemia episodes over the past three months was 2 (IQR: 0-5.2). Table 1 shows the socio-demographic and clinical characteristics of the study participants. 

**Table 1:** Clinical characteristics of the study population HbA1c - glycosylated haemoglobin

Characteristics	n (%); median (IQR)
Gener	
Male	14 (33%)
Female	28 (67%)
Age, years	18.5 (16-24)
Duration of diabetes, years	3 (2-0.2)
BMI, kg/m^2^	23.5 (20.7-27.7)
BMI category	
Underweight	2 (5%)
Normal	22 (52%)
Overweight	12 (29%)
Obese	6 (14%)
Fat mass, %	23.9 (12.6-31.6)
Free fat mass, kg	72.4 (64.9-83.0)
Daily insulin dose, UI/kg/day	0.8 (0.7-1.3)
Hypoglycaemia episode, n	2 (0-5.2)
HbA1c, %	8.4 (6.9-11.3)
Glycaemic control	
Good (HbA1c≤7%)	12 (29%)
Poor (HbA1c>7%)	30 (71%)
Basal C-peptide, nmol/L	0.198 (0.066-0.891)
Stimulated C-peptide, nmol/L	0.231 (0.066-2.676)

Residual endogenous insulin secretion and glycaemic control in the study participants

The median basal and stimulated plasma C-peptide levels were 0.198 (0.066-0.891) nmol/L and 0.231 (0.066-2.676) nmol/L, respectively. Stimulated plasma C-peptide levels were detectable (plasma C-peptide ≥0.033 nmol/L) in 25 (60%) participants, and 12 (29%) participants had high residual endogenous insulin secretion levels (stimulated plasma C-peptide ≥0.2 nmol/L).

Participants with high residual endogenous insulin secretion had significantly lower median HbA1c compared to their counterparts with low and absent residual endogenous insulin secretion (HbA1c 7.1% (IQR: 6.1-8.3%) vs 9.1% (IQR: 8.1-12.2%), p=0.01).

The proportion of participants with high endogenous insulin secretion evaluated by stimulated C-peptide was significantly higher in participants with good glycaemic control compared to those with poor glycaemic control (7/12 (58.3%) vs 5/30 (16.7%), p=0.03), as shown in Table 2.

**Table 2:** Distribution of participants according to C-peptide levels and levels of glycaemic control

C-peptide levels (nmol/L)	Good glycaemic control (HbA1c ≤7%), n=12	Poor glycaemic control (HbA1c >7%), n=30	p-value
Basal C-peptide levels, n (%)			
<0.033	4 (33.3)	25 (83.3)	0.006
0.033-0.2	6 (50.0)	4 (13.3)	
≥0.2	2 (16.7)	1 (3.3)	
Stimulated C-peptide levels, n (%)			
<0.033	4 (33.3)	21 (70.0)	0.03
0.033-0.2	1 (8.3)	4 (13.3)	
≥0.2	7 (58.3)	5 (16.7)	

Comparison of participants with and without high residual endogenous insulin secretion 

Table 3 shows the characteristics of those with and without high residual endogenous insulin secretion. Compared with participants without high residual endogenous insulin secretion, those with high residual endogenous insulin secretion had higher median BMI (26.1 (IQR: 25-29.3) vs 22.9 (IQR: 19.4-25.6) kg/m², p=0.02), higher median fat mass (32.8% (IQR: 29.5-37.3) vs 16.8% (IQR: 11.1-23.3), p<0.001), shorter median diabetes duration (2 (IQR: 1-4) vs 6 (IQR: 2-11) years, p=0.009), lower median daily insulin dose (0.7 (IQR: 0.4-0.8) vs 0.9 (IQR: 0.7-1.3) IU/kg/day, p=0.008), and fewer median hypoglycaemia episodes (0 vs 4, p=0.006).

**Table 3:** Comparison of participants with and without high residual insulin secretion

Characteristics, n (%), median (IQR)	High residual insulin secretion (stimulated C-peptide ≥0.2 nmol/L)		p-value
	Yes, n=12	No, n=30	
Duration of diabetes, years	2 (1-4)	6 (2-11)	0.009
BMI, kg/m^2^	26.1 (25-29.3)	22.9 (19.4-25.6)	0.02
Episodes of hypoglycaemia for the last 3 months, n	0 (0-2)	4 (1-6)	0.006
Fat mass, %	32.8 (29.5-37.3)	16.8 (11.1-23.3)	<0.001
Daily insulin dose, IU/kg/day	0.7 (0.4-0.8)	0.9 (0.7-1.3)	0.008
Insulin therapy regimen			
2 injections/day	4 (33.3)	2 (6.7)	0.04
≥3 injections/day	8 (66.7)	28 (93.3)	

## Discussion

This study demonstrates that in a cohort of young Cameroonians with T1D, residual insulin secretion was relatively common and strongly associated with better glycaemic outcomes. Nearly one-third of participants retained high levels of stimulated C-peptide, and this subgroup exhibited lower HbA1c values, fewer hypoglycaemic episodes, reduced insulin requirements, and higher rates of achieving HbA1c ≤7%. These findings underscore the clinical importance of even modest residual insulin secretion in improving metabolic control.

Our results are consistent with evidence from the Diabetes Control and Complications Trial (DCCT), which showed that residual β-cell function, particularly at higher and sustained levels, was linked to lower HbA1c values and reduced rates of severe hypoglycaemia [6,7]. Similarly, Sørensen et al. observed that children with higher levels of residual secretion had significantly fewer episodes of severe hypoglycaemia compared to those without detectable C-peptide [12]. In long-term studies, Davis et al. and Oram et al. reported that a significant proportion of individuals with long-standing type 1 diabetes retain measurable C-peptide secretion, with those maintaining secretion experiencing better glycaemic control and fewer complications [5,13]. Our findings align with these observations, highlighting that the beneficial effects of residual secretion extend across populations and settings.

The prevalence of residual insulin secretion in our study (60% detectable, 29% high secretion) appears higher than reported in some cohorts with longer disease duration. For example, Davis et al. found that only 29% of participants with a median diabetes duration of 13 years had detectable secretion [5], and Cheng et al. reported 38.5% after more than 10 years of diabetes [4]. By contrast, our cohort had a shorter median duration of three years, which likely explains the higher prevalence. This finding is also consistent with the observation by Harsunen et al., who demonstrated that residual secretion declines with increasing duration of diabetes but can persist for decades in some individuals [14].

Several mechanisms may explain the protective effects of residual insulin secretion. Even low levels of endogenous insulin are thought to buffer against wide glucose fluctuations, reduce glycaemic variability, and mitigate the risk of hypoglycaemia. Endogenous insulin also contributes to improved metabolic efficiency, lowering exogenous insulin needs and reducing the risk of weight gain and insulin resistance. In addition, preserved β-cell function may directly influence long-term complication risk, as suggested by DCCT findings of reduced microvascular complications among participants with residual secretion [6,7].

Our study has important local relevance. Despite free access to insulin and structured care through the Changing Diabetes in Children (CDiC) project, most young people in Cameroon continue to have suboptimal glycaemic control [8,9] and a high mortality rate [15]. Identifying those with little or no residual secretion may help clinicians prioritise intensive education, closer monitoring, and supportive technologies to reduce complications and premature mortality. Moreover, these results reinforce the potential value of incorporating C-peptide testing into routine assessment at diagnosis or a few years after diagnosis, even in resource-constrained settings, to stratify risk and guide individualised care.

This study has limitations. The relatively small sample size limited our ability to conduct stratified analyses across all levels of residual secretion. The diagnosis of type 1 diabetes was based on clinical features rather than autoantibody testing, which may have introduced some misclassification (e.g., inclusion of maturity-onset diabetes of the young, MODY, or type 2 diabetes). However, the young age of onset and insulin dependence in nearly all participants make it unlikely to have significantly affected our conclusions. Also, hypoglycaemia was self-reported by the participants and can potentially be affected by recall bias. Finally, the cross-sectional design precludes assessment of long-term outcomes; longitudinal studies are needed to confirm the durability of residual secretion and its impact on complications in this population. 

## Conclusions

This is the unique study from Cameroon to demonstrate that residual insulin secretion remains present in a substantial proportion of young people with type 1 diabetes and confers significant benefits in terms of glycaemic control. These findings, consistent with international evidence, emphasise the importance of measuring and monitoring residual insulin secretion to better tailor care. Incorporating C-peptide testing at diagnosis or a few years after could help identify vulnerable individuals who may require more intensive interventions to improve outcomes and reduce complications.

## References

[1] American Diabetes Association Professional Practice Committee for Diabetes (2026). 2. Diagnosis and classification of diabetes: standards of care in diabetes-2026. Diabetes Care.

[2] Secrest AM, Washington RE, Orchard TJ (2025). Mortality in Type 1 Diabetes. Diabetes in America, 3rd edition.

[3] Eisenbarth GS (1986). Type I diabetes mellitus. N Engl J Med.

[4] Cheng J, Yin M, Tang X (2021). Residual β-cell function after 10 years of autoimmune type 1 diabetes: prevalence, possible determinants, and implications for metabolism. Ann Transl Med.

[5] Davis AK, DuBose SN, Haller MJ (2015). Prevalence of detectable C-Peptide according to age at diagnosis and duration of type 1 diabetes. Diabetes Care.

[6] The Diabetes Control and Complications Trial Research Group (1998). Effect of intensive therapy on residual beta-cell function in patients with type 1 diabetes in the diabetes control and complications trial. A randomized, controlled trial. Ann Intern Med.

[7] Steffes MW, Sibley S, Jackson M, Thomas W (2003). Beta-cell function and the development of diabetes-related complications in the diabetes control and complications trial. Diabetes Care.

[8] Dehayem MY, Takogue R, Choukem SP (2016). Impact of a pioneer diabetes camp experience on glycemic control among children and adolescents living with type 1 diabetes in sub-Saharan Africa. BMC Endocr Disord.

[9] Djonou C, Tankeu AT, Dehayem MY, Tcheutchoua DN, Mbanya JC, Sobngwi E (2019). Glycemic control and correlates in a group of sub Saharan type 1 diabetes adolescents. BMC Res Notes.

[10] (2025). BMI for age 5-19 years. World Health Organization. https://www.who.int/tools/growth-reference-data-for-5to19-years/indicators/bmi-for-age.

[11] The DCCT Research Group (1987). Effects of age, duration and treatment of insulin-dependent diabetes mellitus on residual beta-cell function: observations during eligibility testing for the Diabetes Control and Complications Trial (DCCT). J Clin Endocrinol Metab.

[12] Sørensen JS, Johannesen J, Pociot F (2013). Residual β-Cell function 3-6 years after onset of type 1 diabetes reduces risk of severe hypoglycemia in children and adolescents. Diabetes Care.

[13] Oram RA, McDonald TJ, Shields BM (2015). Most people with long-duration type 1 diabetes in a large population-based study are insulin microsecretors. Diabetes Care.

[14] Harsunen M, Haukka J, Harjutsalo V (2023). Residual insulin secretion in individuals with type 1 diabetes in Finland: longitudinal and cross-sectional analyses. Lancet Diabetes Endocrinol.

[15] Katte JC, Gwan E, Feuatsap CK, Kamdem L, Chetcha A, Dehayem MY (2025). Mortality and low C-peptide levels in children and young people diagnosed with type 1 diabetes in Cameroon: a case series from the young-onset diabetes in sub-Saharan Africa study. Pan Afr Med J.

